# Action Recognition by an Attention-Aware Temporal Weighted Convolutional Neural Network

**DOI:** 10.3390/s18071979

**Published:** 2018-06-21

**Authors:** Le Wang, Jinliang Zang, Qilin Zhang, Zhenxing Niu, Gang Hua, Nanning Zheng

**Affiliations:** 1 Institute of Artificial Intelligence and Robotics, Xi’an Jiaotong University, Xi’an 710049, China; zjl19920904@stu.xjtu.edu.cn (J.Z.); nnzheng@xjtu.edu.cn (N.Z.); 2 HERE Technologies, Chicago, IL 60606, USA; qilin.zhang@here.com; 3 Alibaba Group, Hangzhou 311121, China; zhenxing.nzx@alibaba-inc.com; 4 Microsoft Research, Redmond, WA 98052, USA; ganghua@microsoft.com

**Keywords:** action recognition, attention model, convolutional neural netwoks, video-level prediction, temporal weighting

## Abstract

Research in human action recognition has accelerated significantly since the introduction of powerful machine learning tools such as Convolutional Neural Networks (CNNs). However, effective and efficient methods for incorporation of temporal information into CNNs are still being actively explored in the recent literature. Motivated by the popular recurrent attention models in the research area of natural language processing, we propose the Attention-aware Temporal Weighted CNN (ATW CNN) for action recognition in videos, which embeds a visual attention model into a temporal weighted multi-stream CNN. This attention model is simply implemented as temporal weighting yet it effectively boosts the recognition performance of video representations. Besides, each stream in the proposed ATW CNN framework is capable of end-to-end training, with both network parameters and temporal weights optimized by stochastic gradient descent (SGD) with back-propagation. Our experimental results on the UCF-101 and HMDB-51 datasets showed that the proposed attention mechanism contributes substantially to the performance gains with the more discriminative snippets by focusing on more relevant video segments.

## 1. Introduction

Action recognition and activity understanding in videos are imperative elements of computer vision research. Over the last few years, deep learning techniques dramatically revolutionized research areas such as image classification [1,2], object segmentation [3,4,5]. Likewise, Convolutional Neural Networks (CNNs) and Recurrent Neural Networks (RNNs) have been popular in the video classification and detection task [6,7,8,9,10,11,12,13,14,15,16,17]. However, various network architectures have been proposed with different strategies on the incorporation of video temporal information. However, despite all these variations, their performance improvements over the finetuned image classification network are still relatively small.

Unlike image classification, the most distinctive property of video data is the variable-length. While images can be readily resized to the same spatial resolution, it is difficult to subsample videos temporally. Therefore, it is difficult for the early 3D convolution neural networks (3D CNNs) [18] to achieve action recognition performance on par with the sophisticated hand-crafted improved Dense Trajectory (iDT) [19] representations.

In addition, some of the legacy action recognition datasets (e.g., KTH [20]) only contain repetitive and transient actions, which are rarely seen in everyday life and therefore have limited practical applications. With more realistic actions included (with complex actions, background clutter and long temporal duration), the more recent action recognition datasets, daily lives videos (UCF-101 [21]) and isolated activities in movies (HMDB-51 [22]), offer much more realistic challenges to evaluate modern action recognition algorithms. Therefore, all experimental results in this paper are based on the UCF-101 and HMDB-51 datasets.

Previous multi-stream architecture, such as the two-stream CNN [6], suffers from a common drawback, their spatial CNN stream is solely based on a single image randomly selected from the entire video, rather than a sequence of video frames. For complicated activities and relatively long action videos (such as the ones in the UCF-101 and HMDB-51 datasets), viewpoint variations and background clutter could significantly complicate the representation of the video from a single randomly sampled video frame. A recent remedy was proposed in the Temporal Segment Network (TSN) [8] with a fusion step which incorporates multiple snippets.

Inspired by the success of the attention model widely used in natural language processing [23] and image caption generation [24], the Attention-aware Temporal Weighted CNN (ATW CNN) is proposed in this paper, to further boost the performance of action recognition by introducting benign competition mechanism between video snippets. The attention mechanism is implemented via temporal weighting: instead of processing all sampled frames equally, the temporal weighting mechanism automatically focuses more heavily on the semantically critical segments, which could lead to reduced noise (Video frames with too many clutter or from unrepresentative viewpoints are less accounted for). In addition, unlike prior pose-based CNN (P-CNN) [12] which requires additional manual labeling of human pose, a soft attention model is incorporated into the proposed ATW CNN, where such additional labeling is eliminated. Each stream of the proposed ATW CNN can be readily trained end-to-end with stochastic gradient descent (SGD) with back-propagation using only existing dataset labels. We perform extensive comparisons to evaluate the action recognition performance of the proposed ATW CNN against state-of-the-art methods with both qualitative and quantitative results on two benchmark action recognition datasets, i.e., the HMDB-51 [22] and UCF-101 [21] datasets. Furthermore, to better understand the contributions of different components of our proposed method, we conduct extensive ablation studies on the proposed method. It is verified that our method compares favorably with the state-of-the-art methods, and has the ability to identify temporally long-range multi-stage actions in long videos.

The major contributions of this paper can be summarized as follows.

- An effective long-range attention mechanism simply implemented by temporal weighting;
- Each stream of the proposed ATW CNN can be optimized end-to-end, without requiring additional labeling;
- State-of-the-art recognition performance is achieved on two public datasets, i.e., the HMDB-51 [22] and UCF-101 [21] datasets.

This paper is an extension to its conference version [25] with reorganized and more comprehensive details of our work, including additional details in problem formulation and implementation, a fully revamped experimental section with new experiments and discussions, and a more comprehensive section on related works with additional representative publications.

The remainder of the paper is organized as follows. In Section 2, a brief review of related works is provided. In Section 3, we formulate the video representations, the proposed ATW CNN and provide implementation details of it. Section 4 contains multiple experimental results and corresponding discussions. Finally, the paper is concluded in Section 5.

## 2. Related Works

Human action recognition has been studied for decades, which is challenging partially due to large intraclass variations in appearance of motions and camera settings, etc. Before the emergence of deep learning, handcrafted action representations were in the prime position along during the progress of action recognition. There were usually two major steps, including the action representation extraction and the classifier training. Among them, many methods are based on spatio-temporal interest point detection. For instance, Laptev et al. [26] extended the Harris and Förstner spatial interest points to the space-time interest points. Lately, trajectory-based action representations became the dominant handcrafted action representations, which are obtained by tracking human body joints throughout the action videos. For example, Wang et al. [19,27] used such trajectories as motion representation by tracking densely sampled points from optical flow. Peng et al. [28] employed local features (bag of visual words) for video representation. Besides, there were also methods leveraging global representations. For example, Shao et al. [29] introduced a spatio-temporal Laplacian pyramid coding method for action representation.

In the past few years, CNN-based techniques have revolutionized the image/video understanding [6,7,8,9,10,13,18,30,31,32]. Per the data types used for action recognition, deep neural networks-based methods can be categorized into two groups: (1) RGBD camera-based action recognition, usually with skeleton data and depth/3D point clouds information [12,33,34]; (2) conventional video camera-based action recognition.

RGBD camera-based action recognition offers 3D information, which is a valuable addition to the conventional RGB channels. Such datasets are usually captured by the Microsoft Xbox One Kinect Cameras, such as the Kinetics dataset [12]. Despite its obvious advantage, there are some limiting factors which restrict such model from wide applications. RGBD video datasets are relatively new and labelled ones are not always readily available. A huge backlog of videos captured by conventional RGB camcorders cannot be parsed by such methods due to modality mismatch [35,36,37]. In addition, pure pose/skeleton-based pipelines rarely achieve recognition accuracy on par with RGB video frame-based pipelines [38,39], making them more suitable for an auxiliary system to existing ones.

Inspired by the success of computer vision with still RGB images, many researchers have proposed numerous methods for the conventional RGB video camera-based action recognition. Deep learning-based action recognition methods can be divided into four major categories.

**Figure 1:**
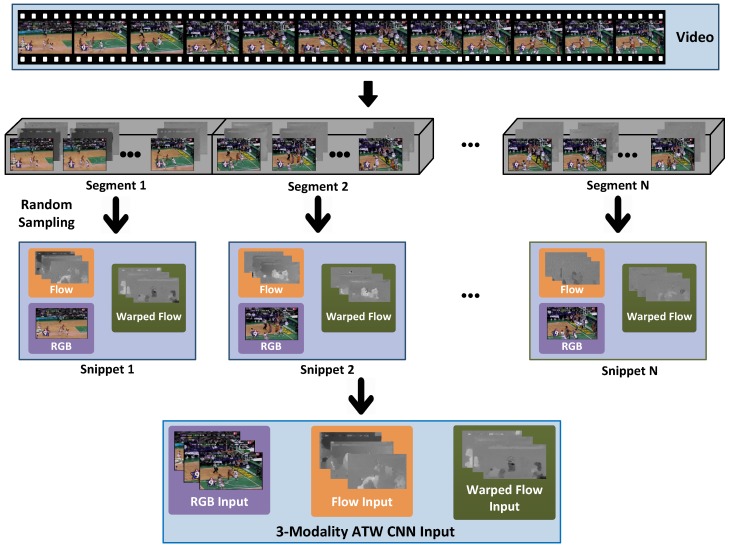
Snippet generation with a fixed target number (*N*) of chunks. A video is evenly portioned into *N* non-overlapping segments. Each segment contains approximately the same number of video frames. As shown above, two additional modalities derived from RGB video frames are also included, i.e., optical flows and warped optical flows. RGB, optical flow and warped optical flow images sampled from the same segment are grouped in a snippet. We put together the randomly sampled video frames and their corresponding optical flows and warped optical flows respectively as the input of the three stream of the proposed ATW CNN.

3D CNNs-based methods. Ji et al. [18] extended regular 2D CNN to 3D, with promising performances achieved on small video datasets. Tran et al. [11] modified traditional 2D convolution kernels and proposed the 3D CNNs for spatio-temporal feature extraction. Sun et al. [40] proposed a cascaded deep architecture which can learn effective spatio-temporal features. Recently, Carreira et al. [9] proposed a new inflated 3D CNN model based on 2D CNNs inflation.
Two-stream CNN-based methods. Simonyan et al. [6] proposed the two-stream CNN by parsing a stack of optical flow images along with RGB images, with each stream being a regular 2D CNN. Since then, optical flow is routinely used as the secondary modality for action recognition. Karpathy et al. [30] studied three fusion strategies (early fusion, late fusion and slow fusion) for the connectivity of streams, which offered a promising way of speeding up the training. Feichtenhofer et al. [13] discovered one of the limiting factors in the two-stream CNN architecture, i.e., only a single frame is sampled from a video as the RGB stream input.
RNN-based methods. Donahue et al. [10] proposed a recurrent architecture (LRCN) to boost the temporal discretion, arguing that temporal discretion via LRCN is critical to action recognition because consecutive video frames often incur redundancies and noises. Ng et al. [7] explored various convolutional temporal feature pooling architectures and connected long-short temporal memory (LSTM) to visual geometry group-16 (VGG-16) networks. The memory cells of LSTM can hold hidden states, and thus can accommodate long-range temporal information. Srivastava et al. [41] used an encoder LSTM to map an input video sequence into a fixed length representation. Mahasseni et al. [42] used LSTM with CNN for action recognition in videos.
Hybrid model-based methods. Hybrid methods incorporate both conventional wisdom and deep learning for action recognition [28,43,44]. Some recent literatures emphasized on new architectures with special considerations for temporal discretion [8,14,45,46,47]. Wang et al. [43] presented the trajectory-pooled deep-convolutional descriptor for video representation. Varol et al. [48] introduced a video representation by using neural networks with long-term temporal convolutions. Apart from these, Zhu et al. [49] proposed a deep framework by using instance learning to identify key volumes and to simultaneously reduce redundancies. Wang et al. [50] proposed a multi-level video representation by stacking the activations of motion features, atoms, and phrases. Fernando et al. [51] introduced a ranking function and used its parameters as video representation. Ni et al. [52] proposed to mine discriminative groups of dense trajectories, which can highlight more discriminative action representation. Wang et al. [8] proposed a video-level framework that aims at exploiting long-term temporal structures for action recognition. Specifically, snippets are multi-modal data randomly sampled from non-overlapping video segments, as shown in Figure 1. Typically a video is divided into 1 to 8 segments. Segments are typically much longer than “clips” used by 3D CNN literature, e.g., the 16-frame clip in 3D CNNs [11].

## 3. Problem Formulation

In this section, the temporally structured video representation model is introduced first, followed by the formulation of the attention-aware temporal weighted convolutional neural network (ATW CNN). After these formulations, the implementation details of the proposed ATW CNN is presented.

### 3.1. Temporally Structured Representation of Action

How do various CNN-based architectures incorporate the capacity to extract semantic information in the time domain? According to the previous two-stream CNN [6] literature, there are generally 3 sampling strategies:

- dense sampling in the time domain, the inputs of the network are consecutive video frames covering the entire video;
- spare sampling one frame out of τ (τ≥2) frames, i.e., frames at time instants 0,t,t+τ,t+2τ,⋯,t+Nτ are sampled;
- with a target number of *N* segments (typical *N* values are from 1 to 8.), non-overlapping segments are obtained by evenly partition the video into *N* such chunks, as illustrated in Figure 1.

As noted by [8,10,13], the dense temporal sampling scheme is suboptimal, with consecutive video frames containing redundant and maybe irrelevant information, recognition performance is likely to be compromised. For the sparse sampling strategy with τ intervals, the choice of τ is a non-trivial problem. With τ too small, it degrades to the dense sampling; with τ too large, some critical discriminative information might get lost. Therefore, the third sampling scheme with fixed target segments is arguably the advisable choice, given the segment number *N* is reasonably chosen.

Suppose a video *V* is equally partitioned into *N* segments, i.e., $V={\left\{S_{k}\right\}}_{k=1}^{N}$, where $S_{k}$ is the *k*-th segment. Inspired by [6,8,53], multi-modality processing is validated to be beneficial. Therefore, our proposed ATW CNN includes three modalities, i.e., RGB video frame, optical flow image and warped optical flow image (as in [19], warped optical flow is obtained by compensating camera motion by an estimated homography matrix), and the combination of them has been proved to be very effective.

In the traditional two-stream structure [6], the dense optical flow representing motion information serves as a supplement to per-frame RGB features, and it is validated to be important and useful for the action recognition task. Moreover, warped optical flow is proposed in iDT [19] to reduce the effect of camera motion on optical flow calculations by estimating the homography matrix. Inspired by these, we further employ the warped optical flow as an additional modality to supplement the RGB and the optical flow inputs, as shown in Figure 1. Thanks to its robustness to camera motion, warped optical flow focuses precisely on human motions, and can contribute to better action recognition performance.

**Figure 2:**
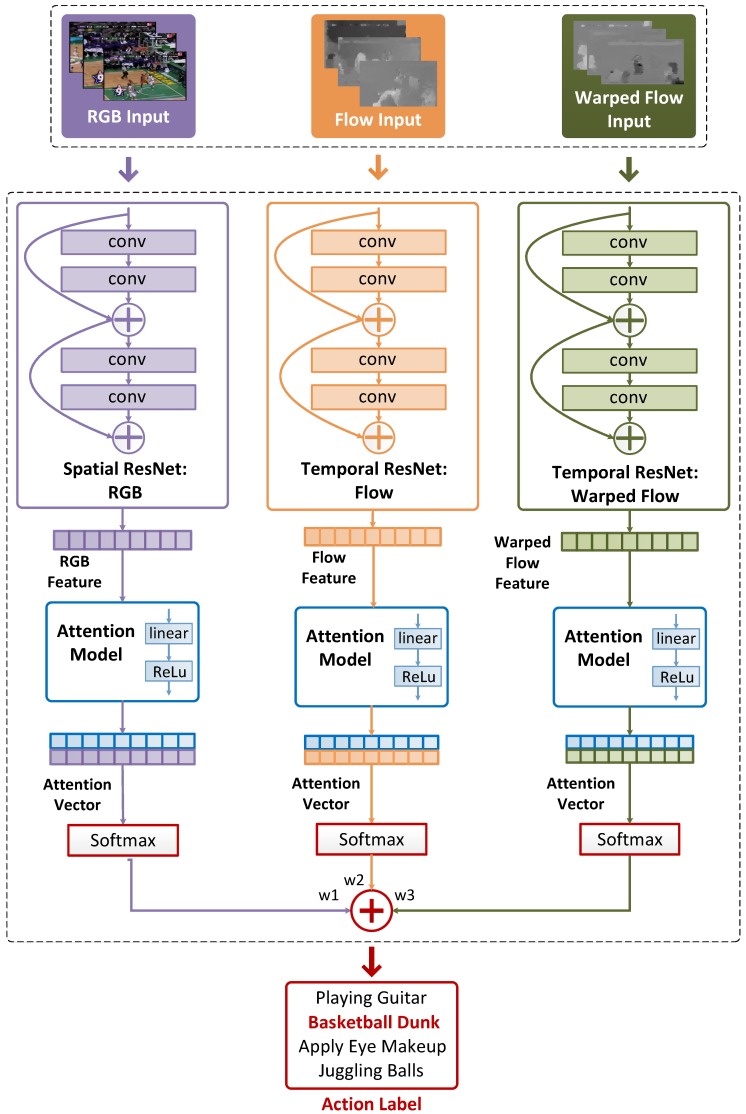
Proposed ATW CNN architecture. Three CNN streams are used to process spatial RGB images, temporal optical flow images, and temporal warped optical flow images, respectively. An attention model is employed to assign temporal weights between snippets for each stream/modality. Weighted sum is used to fuse predictions from the three streams/modalities.

One RGB video frame, five optical flow images and five warped optical flow images are randomly sampled from each segment $S_{k}$ (as illustrated in Figure 1), and they are used as the inputs to the spatial RGB residual convolutional neural networks (ResNet) stream, temporal flow ResNet stream, and temporal warped flow ResNet stream, respectively. RGB, optical flow and warped optical flow images sampled from the same video segment are grouped in a snippet. Each snippet is processed by the proposed 3-stream ATW CNN, and then a per-snippet action probability is obtained, as illustrated in Figure 2. After processing all snippets, a series of temporal weights are learned by the attention model, which are used to fuse per-snippet probabilities into video-level predictions. We proceed to introduce the proposed ATW CNN for action recognition immediately below.

### 3.2. Attention-Aware Temporal Weighted Convolutional Neural Network

The architecture of the proposed ATW CNN for action recognition is presented in Figure 2. During the training phase, every labeled input video *V* is uniformly partitioned into *N* segments, i.e., $V={\left\{S_{i}\right\}}_{i=1}^{N}$, where $S_{i}$ is the *i*-th segment. For each segment $S_{i}$, one RGB video frame, five optical flow images and five warped optical flow images are randomly sampled, as illustrated in Figure 1. Assume $S_{i}$ is represented by three modalities, i.e., $S_{i}={\left\{M_{i}^{RGB},M_{i}^{F},M_{i}^{WF},y\right\}}_{i=1}^{N}$, where $M_{i}^{RGB},M_{i}^{F},M_{i}^{WF}$ respectively represent the RGB, optical flow and warped optical flow images from the *i*-th snippet, with *y* being the corresponding training label. We aggregate the sampled RGB frame and its corresponding optical flows and warped optical flows into a snippet. A series of such snippets are fed to the proposed ATW CNN for training.

ATW CNN aims at the automatic selection of the semantically dominant snippets and the designation of large attention weights to them via the temporal visual attention module. For each modality, it comprises a base CNN stream and a temporal attention model. We choose the ResNet-101 [2] as our base CNN, which is pretrained on the ImageNet dataset [54]. However, our proposed ATW CNN is not limited to any specific CNN network design, and one can choose alternative base CNNs such as the batch normalization (BN)-Inception [55]. Each of the three CNN streams ($C_{RGB}$, $C_{F}$ and $C_{WF}$) maps its corresponding input to a feature vector as

(1) $$\begin{array}{c} C_{RGB}\left(M_{i}^{RGB}\right)=a_{i}^{RGB},\\ C_{F}\left(M_{i}^{F}\right)=a_{i}^{F},\\ C_{WF}\left(M_{i}^{WF}\right)=a_{i}^{WF}, \end{array}$$

where $a_{i}^{RGB}$, $a_{i}^{F}$ and $a_{i}^{WF}$ denote action feature vectors, i.e., the output of the 2nd fully-connected layer of the ResNet before the softmax layer. These action feature vectors are fed into their respective temporal attention models. The attention models in all three streams/modalities are identical in network design, and for notational simplicity, we temporarily drop the superscripts of $a_{i}^{RGB}$, $a_{i}^{F}$ and $a_{i}^{WF}$ and use $a_{i}$ to represent any one of them. A raw attention $e_{i}$, i=1,⋯,N is computed for each snippet in the video with the attention model $f_{att}$ via a multi-layer perceptron conditioned on the fully-connected output of the attention layer ( i.e., $w_{att}$ and ($W_{\mathrm{att}},b_{\mathrm{att}}$) are the parameters of the attention layer) as

(2) $$\begin{array}{c} w_{att}=ReLU\left(W_{\mathrm{att}}a_{i}+b_{\mathrm{att}}\right),\\ e_{i}=f_{att}\left(w_{att},a_{i}\right)=w_{att}^{T}a_{i}. \end{array}$$

Subsequently, they are normalized by a softmax function to guarantee positiveness and unit sum ($\sum_{i=1}^{N}w_{i}=1$) as

(3) $$w_{i}=\frac{\exp e_{i}}{\sum_{j=1}^{N}\exp e_{j}},$$

and the obtained weight $w_{i}$ is used to characterize the semantic relevance of the *i*-th snippet, i.e., the temporal attention weight for the *i*-th snippet with respect to the entire video (specifically, a degenerate case appears if $w_{i}\equiv \frac{1}{N}$, ∀N=1,⋯,N, which means all snippets are deemed “equally important” ).

Afterwards, the attention mechanism φ is implemented with a linear layer followed by a rectifier (ReLU), which serves as a temporal weighting function that aggregates all the per-snippet prediction probabilities with the non-negative weights ${\left\{w_{i}\right\}}_{i=1}^{N}$,

(4) $$\begin{array}{c} A_{att}^{RGB}=\varphi \left(a_{1}^{RGB},\cdots ,a_{N}^{RGB}\right)=softmax\left(\sum_{i=1}^{N}w_{i}^{RGB}a_{i}^{RGB}\right),\\ A_{att}^{F}=\varphi \left(a_{1}^{F},\cdots ,a_{N}^{F}\right)=softmax\left(\sum_{i=1}^{N}w_{i}^{F}a_{i}^{F}\right),\\ A_{att}^{WF}=\varphi \left(a_{1}^{WF},\cdots ,a_{N}^{WF}\right)=softmax\left(\sum_{i=1}^{N}w_{i}^{WF}a_{i}^{WF}\right). \end{array}$$

Finally, the per-video predictions $A_{att}^{fused}$ are obtained with the three attention vectors $A_{att}^{RGB}$, $A_{att}^{F}$, and $A_{att}^{WF}$ fused by fixed weight averaging,

(5) $$A_{att}^{fused}=w_{1}A_{att}^{RGB}+w_{2}A_{att}^{F}+w_{3}A_{att}^{WF}.$$

The entire proposed ATW CNN model is differentiable with the attention model directly embedded, therefore, the gradients of the loss function can freely back-propagate, and the entire framework can be trained end-to-end.

### 3.3. Implementation Details of ATW CNN

During the training phase, images from all three modalities (RGB, optical flow and warped optical flow) are cropped to 224×224. We choose such input resolution for easier reuse of existing image classification network designs without requiring retraining network parameters from scratch. We employ cross modality pre-training [8]. Firstly, the spatial stream (ResNet or BN-Inception) is pre-trained on the ImageNet image classification dataset [54]. Subsequently, these pre-trained weights are used to initialize all 3 streams in the ATW CNN. Each stream of the proposed ATW CNN is trained independently. We use a single frame (1) and a stack of (5) consecutive (warped) optical flow frame as inputs. Based on the standard cross-entropy loss function, the SGD algorithm is used with a mini-batch size of 128. We use an initial learning rate of 0.001 for the spatial stream and 0.005 for both temporal streams. For spatial stream, the learning rate is multiplied by a factor of 0.1 every 2000 iterations. For both temporal streams, the learning rate decay is divided into 2 stages. Learning rates are multiplied by 0.1 at iteration 12,000 and iteration 18,000. Multi-stage training strategy promotes better practical convergence and mitigates over-fitting. All momentums are fixed at 0.9. As the action recognition datasets are significantly smaller than image classification datasets and the risk of overfitting is higher, data augmentation is crucial for the performance of our network architecture. During the training we use random cropping proximate to the image frame corners and scale jittering. We randomly extract four regions that are corners or the center of the image. The width and height of the cropped regions are randomly selected from {168,192,224,256}. The specific random cropping and jittering contribute to more robust understanding of scene semantics by mitigating the implicit attention bias towards the frame centers.

During the testing phase, a fixed number of snippets (80 in our experiments) are uniformly sampled from each video. We use weighted average fusion (with empirically determined fixed weights 1, 1 and 0.5 for the spatial stream, optical flow temporal stream, and warped optical flow temporal stream, respectively) to generate a per-video prediction. The test time for each video of RGB data is approximately 0.64 s, and each video of flow or warped flow data is approximately 1.17 s.

Pytorch [56] is used in our experiments, and the optical flow and the warped optical flow are implemented in OpenCV (OpenCV foundation, Santa Clara, CA, USA) with CUDA 8.0. To speed up training process, 2 NVIDIA Titan Xp GPUs (NVIDIA Corporation, Santa Clara, CA, USA) are used.

## 4. Experiments and Discussions

In this section, we first briefly introduce the two action recognition video datasets, i.e., UCF-101 [21] and HMDB-51 [22]. Subsequently, a series of comparative experiments are conducted and performance evaluation of the proposed ATW CNN against popular baselines are carried out.

### 4.1. Trimmed Action Datasets

In a trimmed action recognition video dataset, each video contains actions of only one action label. The scene is relatively simple and there are generally no more than two people present in the scene. The UCF-101 [21] and HMDB-51 [22] datasets are two such action recognition benchmarks.

The UCF-101 dataset is one of the largest action recognition datasets containing 13,320 YouTube video clips of 101 action categories, including “human-object interaction”, “body-motion”, “human-human interaction”, “playing musical instruments” and “sports”. Among them, sports related videos account for the majority of the dataset. Each video lasts approximately 2 to 15 s.

The HMDB-51 dataset is a highly challenging dataset with 6766 video clips (3570 training and 1530 testing videos) in 51 categories. The videos are collected from various sources, mostly from movies, and the other from websites such as the Prelinger archive, YouTube and Google. The action categories include “general facial actions”, “facial actions with object manipulation”, “general body movements”, “body movements with object interaction” and “body movements for human interaction”. Compared with UCF-101, video clips in HMDB-51 are more challenging because they are generally more representative of the complexity of real-world actions. Evaluation on these two trimmed datasets is performed with average accuracy as the criterion.

### 4.2. Video Frame Sampling Strategies

In this subsection, we compare different snippet sampling strategies in Section 3.1 so that an optimal one can be used in our proposed ATW CNN. There are three choices of sampling strategies:

- dense sampling in time domain;
- interval sampling (1 sample every τ frames);
- given the predefined total number of segments *N*, each video is evenly partitioned into *N* non-overlapping segments (denoted as “Uniform Segmentation” in Table 1).

For fair comparison, we choose the VGG-16 architecture [1] and the UCF-101 dataset (split1) in this experiment, and follow the suggestions in [13] and let τ=15 and N=4. No attention mechanism is used to eliminate possible interferences. The VGG-16 architecture is used for easier and faster training. Three types of network are used, RGB image-based VGG-16, optical flow-based VGG-16, and a two-stream CNN with both the RGB VGG-16 and optical flow VGG-16. The overall classification accuracies are summarized in Table 1.

The dense sampling strategy offers significantly worse performance for the RGB VGG-16 network, and it deteriorates the performance of optical flow VGG-16 and 2-stream RGB + flow VGG-16 so much that more than half of testing samples are misclassified. The interval sampling scheme is empirically verified to be optimal for the RGB image-based VGG-16 network, but the “uniform segmentation” strategy is proved to be better for both the optical flow VGG-16 network and the 2-stream RGB + Flow VGG-16 network. Considering the similarity between the proposed 3-stream ATW CNN and the 2-stream RGB + Flow VGG-16 network, the “uniform segmentation” strategy is chosen and utilized throughout the remainder of the paper.

### 4.3. Comparison with Different Consensus Functions

In the subsection, we explore the effects of two alternative consensus functions against the proposed attention model, i.e., the max segmental consensus and the average segmental consensus against the proposed attention model. The max and average segmental consensus functions are implemented by replacing the “Attention Model” module in Figure 2 by a “MAX” and “AVERAGE” operator, respectively. For fair comparison and faster network training, the traditional BN-Inception [55] network architecture is used with *N* in Equation (1) fixed at 4 and evaluated on the first split of UCF-101. Three types of network are included in this comparison, including the single stream RGB image-based BN-Inception net, the single stream optical flow image-based BN-Inception net and the combined 2-stream RGB+Flow image-based BN-Inception net. The experimental results are summarized in Table 2 in terms of classification accuracy. The average segmental consensus function slightly outperforms the max counterpart with all 3 networks, but the best results are obtained by the proposed attention model, which significantly improves the efficacy of temporal/segmental consensus fusion across all 3 networks.

### 4.4. Choice of Segment Number *N* in Attention Model

In this subsection, different choices of the number of segments (*N* in Equation (1)) are empirically tested and the results are summarized in Table 3 in terms of classification accuracy. For fair comparison and faster network training, the proposed attention model is incorporated into a simpler single stream, RGB image-based network (with the BN-Inception [55] architecture) and evaluated on the first splits of both the UCF-101 and HMDB-51 datasets.

With small segment numbers (e.g., N<3), the proposed attention model is significantly oversimplified and even degenerates (if N=1). With the appropriate choice of N=4, the attention model achieves the optimal 85.80% accuracy on the UCF-101 (split 1) and the optimal 53.88% accuracy on the HMDB-51 (split 1). We note that excessively large segment number leads to slight performance degradations as shown in Table 3. In addition, excessively large segment number incurs larger computational cost, consumes more GPU memory and slows down the overall training process. Based on such observations, the total segment number *N* is fixed at 4 for the proposed ATW CNN for the remainder of the paper.

### 4.5. Activation Function Selection and Parameter Initialization in Attention Layers

Proper selection of the activation function and the initialization strategy are also important for achieving the optimal recognition performance. For a fair comparison and faster network training, the proposed attention model is incorporated into two simpler networks, both of which are single stream nets based on the BN-Inception [55] architecture. One of them uses RGB images as input, and the other uses optical flow images as inputs, and they are denoted as RGB BN-Inception Net and Optical Flow BN-Inception Net in Table 4 and Table 5.

To find the optimal activation functions, we test multiple selections of common activation functions in the attention layer and the respective classification accuracies on the first split of UCF-101 dataset are summarized in Table 4. ReLU is found to be marginally better than the sigmoid and the hyperbolic tangent (tanh), therefore we choose ReLU as the attention layer activation function in our proposed ATW CNN.

Different initialization strategies also contribute to performance differences. Three common initialization strategies for the weights in the proposed attention layer are empirically tested on the first split of the UCF-101 dataset, including

- all weights $w_{i}$ set to 1 and biases $b_{i}$ set to 0;
- all weights $w_{i}$ set to $\frac{1}{N}$ and bias $b_{i}$ set to 0;
- random initialization based on standard normal distribution (0 mean and 0.001 standard deviation) for both $w_{i}$ and $b_{i}$.

As shown in Table 5, the standard normal distribution-based random initialization is optimal for both the RGB BN-Inception net and the optical flow BN-Inception net. Therefore, we choose this initialization for our proposed ATW CNN.

### 4.6. Comparison with State-Of-The-Arts

To fully evaluate the performance of the proposed ATW CNN, we compare it with 14 existing state-of-the-art action recognition methods [6,8,11,40,43,48,49,51,52]. With all design choices empirically determined in previous experiments (including video frame sampling strategy, segmental consensus function, appropriate segment number, activation function and parameter initialization), we implement the complete version of the proposed ATW CNN with three streams of ResNet-101 [2] and evaluated the ATW CNN on the complete HMDB-51 and UCF-101 datasets. The spatial RGB ResNet stream is pre-trained on the ImageNet dataset, and the two temporal streams (temporal optical flow stream and the temporal warped optical flow stream) are both initialized by cross-modality pretraining [8]. A simple but effective weighted average opteration is used to fuse the outputs from the three stream ($w_{1}=1$, $w_{2}=1$, $w_{3}=0.5$ as in Figure 2 for the spatial stream, optical flow stream, and warped optical flow stream, respectively).

The action recognition accuracies of the proposed ATW CNN and 14 competing methods [6,8,11,40,43,48,49,51,52] on the HMDB-51 [22] and UCF-101 [21] datasets are summarized in Table 5, in which the results of all the competing methods are taken from respective publications. This comparison shows that the proposed ATW CNN outperforms all 14 recent state-of-the-art methods on both the HMDB-51 [22] and UCF-101 [21] datasets, which validates the efficacy of the proposed attention model and the ATW CNN.

### 4.7. Visualization

**Figure 3:**
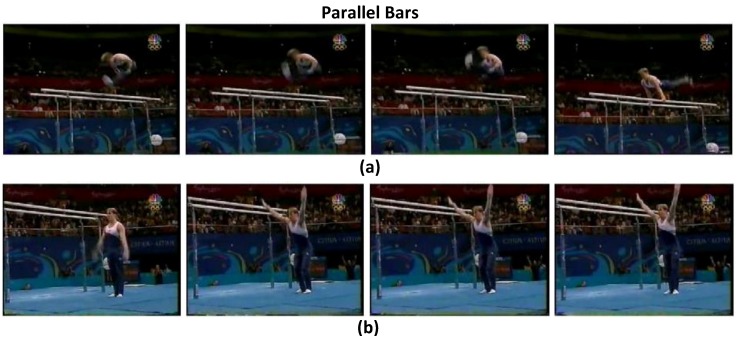
(**a**) The highest ranking four frames in terms of attention weights, in which the athlete is performing on the parallel bars. (**b**) The lowest ranking four frames in terms of attention weights, in which the athlete is standing on the ground.

**Figure 4:**
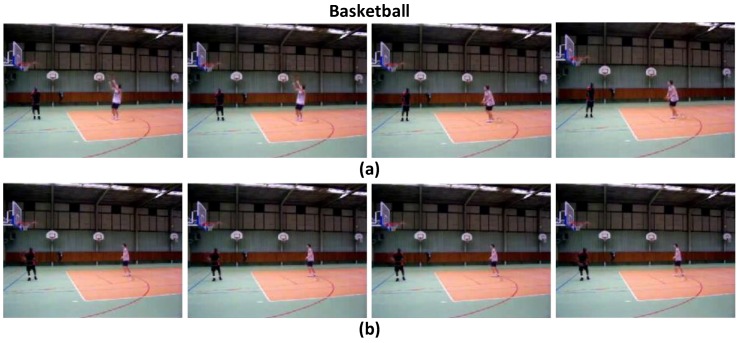
(**a**) The highest ranking four frames in terms of attention weights, in which the athlete is shooting jump. (**b**) The lowest ranking four frames in terms of attention weights, in which the athlete exhibits before or after the shooting.

**Figure 5:**
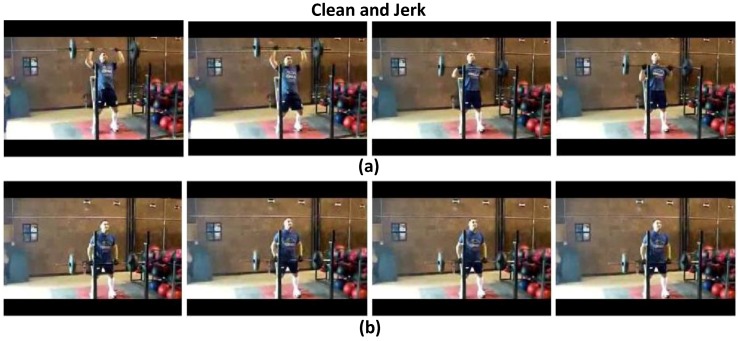
(**a**) The highest ranking four frames in terms of attention weights, in which the athlete is lifting the barbell. (**b**) The lowest ranking four frames in terms of attention weights, in which the athlete is putting down the barbell.

**Figure 6:**
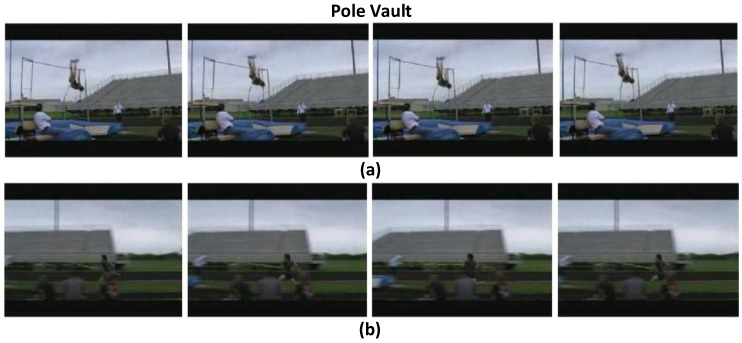
(**a**) The highest ranking four frames in terms of attention weights, in which the athlete is pole-vaulting. (**b**) The lowest ranking four frames in terms of attention weights, , in which the athlete is running up.

To validate the effects of the attention model in the proposed ATW CNN, we visualize the learned temporal visual attention in terms of most relevant and irrelevant video frames, to cast light on how the attention weighting operation interprets the target activity. We present the highest ranking and lowest ranking four video frames in attention weights ($w_{i}$) learned by the proposed attention model in four sample videos of the UCF-101 dataset, i.e., “Parallel Bars" in Figure 3, “Basketball" in Figure 4, “Clean and Jerk" in Figure 5, and “Pole Vault" in Figure 6.

As can be seen in Figure 3, Figure 4, Figure 5 and Figure 6 that, the proposed attention mechanism prioritizes semantically discriminative video frames (the most critical stages) of specific actions by assigning higher attention weight values on them. Correspondingly, less informative frames are assigned with low attention weights as expected. For example, in Figure 3, frames containing the athlete actually performing on the parallel bars are assigned higher attention weights than the ones where the athlete is standing on the ground. Interestingly, our proposed ATW CNN assigns lower attention weights to video frames containing nuisances such as motion blur, as can be seen in Figure 6, video frames in which the athlete is running up are corrupted by large motion blur caused by camera panning. Such running-up frames are less critical in identifying whether the athlete is pole-vaulting or not, and unsurprisingly they are designated the smallest visual attention weights.

To summarize, the above experimental results on the HMDB-51 [22] and UCF-101 [21] datasets reveal that the proposed attention model contributes substantially to the performance gains, with more discriminative snippets focusing on more relevant video frames. In addition, we observe that the proposed ATW CNN achieves superior performance on short actions and handles longer-range multi-stage actions gracefully, such as the “High Jump", “Pole Vault", and “Basketball Dunk" actions in the UCF-101 dataset.

## 5. Conclusions

We presented the ATW CNN, which is a deep multi-stream neural network that incorporates temporal attention for action recognition. It incorporates visual attention with a series of data-adaptive temporal weights, effectively reducing the side effects of redundant information and noise interference from less relevant video frames. Images from three modalities (RGB, optical flow, and warped optical flow images) are fed to three individual CNN streams, respectively, with respective attention models and a late fusion procedure to induce attention weighting in predictions. We evaluated the proposed ATW CNN on two benchmark action recognition datasets, i.e., the HMDB-51 [22] and UCF-101 [21] datasets. The experimental results validated the efficacy of the proposed ATW CNN method and a series of ablation studies verified the effects of the temporal attention model.

For potential future work, we are planning to further extend the proposed attention model to account for spatial ( i.e., pixel-wise) attention and possibly long-term temporal attention in untrimmed videos based on RNNs and LSTMs.

## Figures and Tables

**Table 1:** Classification accuracies by using different sampling strategies for different CNNs on the UCF-101 dataset (split1).

Strategy	RGB VGG-16	Optical Flow VGG-16	RGB + Flow VGG-16
Dense Sampling	68.4%	42.5%	46.8%
Interval Sampling	82.3%	86.2%	90.6%
Uniform Segmentation	79.8%	86.7%	90.9%

**Table 2:** Classification accuracies by exploring different segmental consensus functions on the UCF-101 dataset (split1).

Consensus Function	RGB BN-Inception	Optical Flow BN-Inception	RGB + Flow BN-Inception
Max	85.0%	86.0%	91.6%
Average	85.0%	87.9%	92.5%
Attention Model	86.7%	88.3%	93.8%

**Table 3:** Classification accuracies by choosing different total segment numbers (*N* in Equation (1)) on a RGB image-based BN-Inception net with the proposed attention model.

Dataset	RGB BN-Inception Net with Proposed Attention Model							
	N = 1	N = 2	N = 3	N = 4	N = 5	N = 6	N = 7	N = 8
UCF-101 (split1)	83.33%	83.89%	84.80%	85.80%	85.29%	85.21%	85.04%	85.55%
HMDB-51 (split1)	50.07%	53.33%	53.01%	53.88%	53.33%	55.36%	53.20%	53.14%

**Table 4:** Classification accuracies by selecting different activation functions for the attention layer on the UCF-101 dataset (split1).

Activation Function	RGB BN-Inception Net	Optical Flow BN-Inception Net
tanh	84.91%	87.64%
Sigmoid	85.29%	87.68%
ReLU	85.80%	88.34%

**Table 5:** Classification accuracies of the proposed ATW CNN and other state-of-the-arts on the UCF-101 dataset and the HMDB-51 dataset.

HMDB-51		UCF-101	
Model	Accuracy	Model	Accuracy
DT [57]	55.9%	DT [57]	83.5%
iDT [19]	57.2%	iDT [19]	85.9%
BoVW [28]	61.1%	BoVW [28]	87.9%
MoFAP [50]	61.7%	MoFAP [50]	88.3%
Composite LSTM [41]	44.1%	LRCN [10]	77.0%
RLSTM [42]	55.3%	RLSTM [42]	86.9%
Two Stream [6]	59.4%	Two Stream [6]	88.0%
VideoDarwin [51]	63.7%	C3D [11]	85.2%
MPR [52]	65.5%	Two stream + LSTM [7]	88.6%
$F_{\mathrm{ST}}\mathrm{CN}$ (SCI fusion) [40]	59.1%	$F_{\mathrm{ST}}\mathrm{CN}$ (SCI fusion) [40]	88.1%
TDD + FV [43]	63.2%	TDD + FV [43]	90.3%
LTC [48]	64.8%	LTC [48]	91.7%
KVMF [49]	63.3%	KVMF [49]	93.1%
TSN (3 modalities) [8]	69.4%	TSN (3 modalities) [8]	93.4%
Proposed ATW CNN	70.5%	Proposed ATW CNN	94.6%

## Abbreviations

The following abbreviations are used in this manuscript:

CNNs	Convolutional Neural Networks
LSTM	Long-Short Temporal Memory
ATW	Attention-based Temporal Weighted
SGD	Stochastic Gradient Descent
RNNs	Recurrent Neural Networks
iDT	improved Dense Trajectory
TSN	Temporal Segment Network
P-CNN	Pose-based Convolutional Neural Network
LRCN	Long-term Recurrent Convolutional Networks
C3D	3D Convolutional Networks
ReLu	Rectified Linear Unit
BN	Batch Normalization
ResNet	Deep Residual Convolutional Neural Networks
VGG	Visual Geometry Group
mAP	mean Average Precision
BoVW	Bag of Visual Words
MoFAP	Motion Features, Atoms, Phrases
$F_{\mathrm{ST}}\mathrm{CN}$	Factorized Spatio-Temporal Convolutional Networks
TDD	Trajectory-Pooled Deep-Convolutional Descriptors
FV	Fisher Vector
LTC	Long-term Temporal Convolutions
KVMF	Key Volume Mining deep Framework
